# Supplementary material for: Time-Related Eating Patterns Are Associated with the Total Daily Intake of Calories and Macronutrients in Day and Night Shift Workers
Source: Nutrients. 2022 May 25;14(11):2202. doi: 10.3390/nu14112202 (PMC9182503; doi:10.3390/nu14112202)
Supplement: Supplementary file 1 [file nutrients-14-02202-s001.zip › nutrients-1688230-supplementary.pdf]

Supplementary Material

Supplementary Material **S**1. Energy and macronutrients distributed in periods according to the shift.

Values of energy/macronutrients per period and the isolated effect of the eating period

- ***Energy:***
  - **Values:** Period 1: 421.5±31.1kcal; Period 2: 965.6±48.4kcal; Period 3: 980.4±68.6kcal; Period 4: 625.0±80.0kcal;
  - **Comparisons:** P2, P3 and P4>P1 (p<0.001; p<0.001; p=0.020, respectively); P2 and P3>P4 (p<0.001 and p=0.001, respectively);

- ***Carbohydrate:***
  - **Values:** Period 1: 199.4±15.9kcal; Period 2: 406.9±25.9kcal; Period 3: 400.0±33.7kcal; Period 4: 269.3±34.8kcal;
  - **Comparisons:** P2 and P3>P1 (p<0.001 for both); P2 and P3>P4 (p=0.002 and p=0.006, respectively);

- ***Fat***:
  - **Values:** Period 1: 169.9±14.8kcal; Period 2: 340.3±24.0kcal; Period 3: 383.7±30.3kcal; Period 4: 227.4±36.4kcal;
  - **Comparisons:** P2 and P3>P1 (p<0.001 for both); P2 and P3>P4 (p=0.009 and p=0.003, respectively);

- ***Protein***:
  - **Values:** Period 1: 51.4±4.6kcal; Period 2: 215.1±11.5kcal; Period 3: 199.8±15.6kcal; Period 4: 149.9±27.7kcal;
  - **Comparisons:** P2, P3 and P4>P1 (p<0.001; p<0.001 and p=0.001, respectively).
